# Comment on 'Addition of ultrasound to mammography in the case of dense breast tissue: systematic review and meta-analysis'

**DOI:** 10.1038/s41416-018-0237-0

**Published:** 2018-11-09

**Authors:** Min Zhu, Ming Li, Jia Liu, Bin Lu

**Affiliations:** 1 Department of Anesthesiology, Zigong Fourth People’s Hospital, Zigong, Sichuan China; 2 Department of Anesthesiology and Translation Neuroscience Center, West China Hospital, Sichuan University, Chengdu, Sichuan China; 3 Department of Anesthesiology, Chengdu Second People’s Hospital, Chengdu, Sichuan China

**Keywords:** Outcomes research, Clinical trials

Sir,

We read with great interest the recent meta- analysis written by Rebolj and colleagues,^1^ which assessed the effect of supplementary ultrasound to mammography in screening women with dense breast tissue on the detection of breast cancer. The authors concluded that supplementary ultrasound screening would be beneficial to increase the sensitivity of breast cancer detection. We appreciate the authors’ thorough analysis, which is a clinically valuable study. However, we have some concerns about the strength of their conclusion owing to some important limitations of the findings in this review.

First, the authors’ search was limited to PubMed. Unfortunately, other international databases such as Embase, CENTRAL databases and Web of Science were not explored. The search strategy may result in the potential for selection bias influencing the generalisability of the study findings, and even incorrect conclusions.^2^ Therefore, an optimised search strategy is necessary to ensure a thorough search of the literature.

Second, high between-study heterogeneity cannot draw a firm conclusion, which stems from a statistical, methodological or clinical aspect.^2^ No matter what the authors adopted, a random-effects model or fixed effect model, the heterogeneity among the studies was prominent for the proportion of total cancers detected only by ultrasound (*I*^2^ > 80%).^1^ Although the authors made effects to find out the sources of the high heterogeneity by subgroup analyses, the results did not appear to explain the variation between studies. Meta-regression could have been conducted to better clarify the factors of variation among the studies, for meta-analyses with a larger number of studies (generally > 10);^3^ there was a sufficient number of studies in this review. Thus, a meta-regression analysis might be more appropriate than a subgroup analysis.

In conclusion, the authors analysed a valuable issue regarding the clinical efficacy on the use of adjunctive ultrasound for screening women with dense breast tissue, but the results of this meta-analysis should be interpreted with caution due to the abovementioned limitations.^3^
